# Novel Giant Magnetoimpedance Magnetic Field Sensor

**DOI:** 10.3390/s20030691

**Published:** 2020-01-27

**Authors:** Piotr Gazda, Roman Szewczyk

**Affiliations:** Institute of Metrology and Biomedical Engineering, Warsaw University of Technology, 02-525 Warsaw, Poland; szewczyk@mchtr.pw.edu.pl

**Keywords:** giant magnetoimpedance, magnetic field sensor, compensation measurements

## Abstract

The idea, design, and tests of the novel GMI sensor are presented, based on the compensation measurement principle, where the local ‘zero-field’ minimum of the double-peak characteristic was utilized as a sensitive null detector. The compensation field was applied in real-time with the help of microprocessor-based, two-step, quasi-Newtonian optimization. The process of material parameters optimization through Joule-annealing of chosen amorphous alloys is described. The presented results of the prototype test unit show linear output characteristic, low measurement uncertainty, and resistance against time and temperature drift.

## 1. Introduction

Sensors for measuring the magnetic field play a key role in many areas of today’s science and technology. The areas of their application include space research [1], military applications and security systems [2,3], high-density magnetic memory [4], non-destructive testing [5], navigation [6], geology [7], medicine [8], current transformers [9], and many others [10]. The current total value of the magnetic field sensor market is estimated at about two billion U.S. dollars a year, of which the largest volume is associated with power equipment automation systems and car automation [11].

One of the effects with great potential in sensor application is the phenomenon of giant magnetoimpedance (GMI). The GMI phenomenon consists of a significant (gigantic) change in the impedance of the high-frequency ferromagnetic AC conductor in the presence of a constant magnetic field [12]. The impedance changes reach several hundred % [13], while the sensitivity reaches 1.5%/A/m [14]. This allows for the development of a high-resolution sensor. The development of GMI magnetic field sensor technology was mainly in the nineties and 2000s [10,15,16,17,18].

The work related to the investigation of the GMI phenomenon is strongly orientated toward the development of material technology: the measurements of the effect in subsequent materials or geometric structures, the improvement of the impedance change rate, or the sensitivity of impedance changes to the applied magnetic field. These studies, initiated 25 years ago [19,20], are chart-topping to this day [21,22,23,24,25,26,27,28]. Most studies have been concerned with amorphous microwires [21,22,23,24,25] subjected to various forms of annealing [22,24,25] and covered with layers reinforcing the effect [23], or in contrast, when the glass coating of the wire is etched [21]. Research has also been carried out using micro-patterned amorphous ribbon [26,27] and thin layers [28]. 

Nevertheless, GMI sensor technology based on various operating principles has also developed over the years. Some have been based on microprocessor processing of the characteristics, mainly on the linear part of the impedance curve. The computational capabilities of the microprocessors of that time were small when compared to current solutions, which is why the prototype designs of GMI sensors were based mainly on analog electronics [10,18,29,30,31]. A quasi-linear relationship occurs for a double-peak curve between the zero magnetizing field and the anisotropy field. Direct measurements of a weak magnetic field using a linear relationship require additional biasing magnetization. It is possible to produce a material with a linear characteristic above the anisotropy field, but it also requires proper biasing for operation. The solution to the problem of shifting the characteristics can be the application of two active GMI elements working in a differential system. Another solution to this problem is the use of a pick-up coil surrounding a GMI element connected to a phase-sensitive amplifier. Then, the ‘negative’ part of the characteristic has negative values, symmetrical to the point (0.0). This allows for linear characteristics to be achieved in the range of −H_k_ (anisotropy field) and +H_k_. Utilizing the GMI effect to construct GMI sensors has been noted [32,33,34,35,36,37], and are used as magnetic memories [34], automotive sensors [35], or biosensors [32,33].

In this paper, a GMI magnetic field sensor was proposed, based on the double-peak characteristics of joule-annealed amorphous ribbons and a novel two-step, microprocessor-based automatic compensation measurement procedure. 

## 2. Materials and Methods

### 2.1. Test Stand for Measuring the Giant Magnetoimpedance Phenomenon

**Figure 1:**
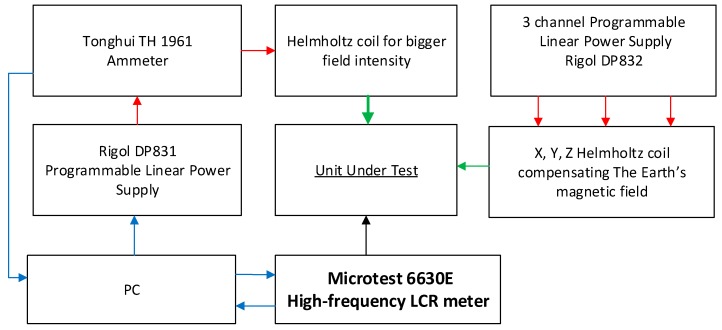
Block diagram of the test station for measuring the Giant Magnetoimpedance phenomenon.

**Figure 2:**
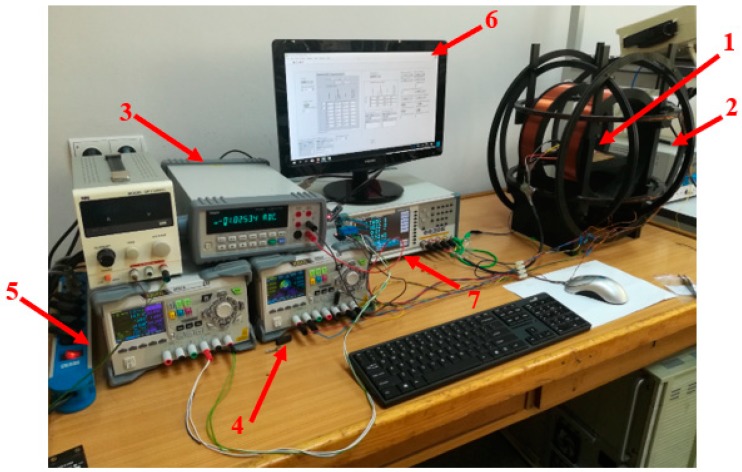
Photograph of the GMI phenomenon measurement station: (1) Unit under test; (2) Helmholtz coils for setting the magnetic field; (3) ammeter; (4) laboratory power supplies for compensating coils; (5) laboratory power supply for the main coil; (6) PC; (7) high-frequency LCR meter.

Research on the GMI phenomenon in the samples produced from ribbons made of amorphous alloys was carried out with the use of the specially developed impedance Z measurement station as a function of an external magnetizing field, H. The block diagram of the developed station is shown in Figure 1, and the photo of the station is shown in Figure 2.

Careful sample mounting ensured the small influence of stress on the results; this is particularly important due to the GMI-related [38] the stress–impedance effect (i.e., a change in the sample impedance under the influence of stress-induced change in magnetic permeability) [39]. This is one of the adverse instances of the significant magnetoelastic effect in amorphous alloys [40,41], which changes the whole magnetization process due to induced anisotropy [42,43], but could be otherwise utilized [44,45]. To control the entire measuring station and acquire the measurement data, a custom application was written in the LabVIEW environment.

### 2.2. Preparation and Investigation of the Sensor Core 

The material analysis was carried out to determine the appropriate material for the core of the developed sensor. For this purpose, samples made of cobalt-based amorphous ribbons were tested. Materials selected for testing were alloys with a saturation magnetostriction close to zero: Co_70_Fe_5_Ni_2_Mo_5_B_3_Si_15_, Co_66_Fe_4_Ni_1_Si_15_B_14_, and Co_67_Fe_3_Cr_3_Si_15_B_12_; and alloys with negative magnetostriction: Co_83,4_Fe_1,4_Mo_1,7_Mn_4,3_Si_7,3_B_1,9_ and Co_87,45_Fe_5,42_Si_3,34_B_3,79_. Samples were 1 mm wide and 50 mm long. The ribbon samples were subjected to the Joule annealing process for an hour in a chamber filled with a protective atmosphere. The annealing current for the subsequent samples ranged from 500 mA to 1100 mA in steps of 100 mA. The annealing current value for the Co_66_Fe_4_Ni_1_Si_15_B_14_ alloy was later further optimized, and additional samples were annealed with a current from 550 mA to 650 mA every 25 mA.

The coefficient of the impedance changes, otherwise known as the GMI coefficient, is defined as:

(1) $$\frac{∆Z}{Z}\;\left(\%\right)\;=\;100\;\cdot \;\frac{Z\left(H\right)-Z\left(H_{max}\right)}{Z\left(H_{max}\right)}$$

**Figure 3:**
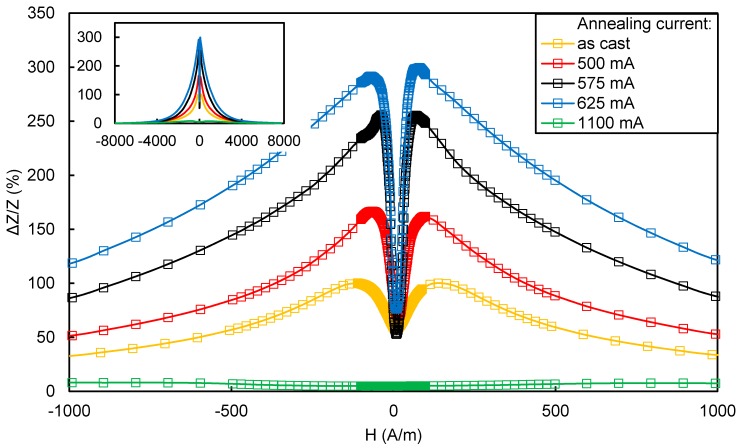
The impedance change characteristics ΔZ as a function of the external magnetizing field H for samples from the amorphous alloy with the composition Co_66_Fe_4_Ni_1_Si_15_B_14_ in the initial state and subjected to the process of thermomagnetic relaxation.

The conducted material research focused on finding a material with the lowest possible H_k_ anisotropy field and a large change in the GMI impedance. The excitation signal had a frequency of 10 MHz. Figure 3 shows the results for the best one: Co_66_Fe_4_Ni_1_Si_15_B_14_ alloy. This alloy has significant lower noise spectrum than Co_70_Fe_5_Ni_2_Mo_5_B_3_Si_15_ [46]. Numerous publications describing the use of the Co_66_Fe_4_Ni_1_Si_15_B_14_ alloy on the fluxgate [47,48] or induction [49] sensor core have confirmed its excellent noise properties. Figure 3 shows only part of the results to make it legible; the inset of Figure 3 indicates the GMI characteristics in the full area of the magnetizing field. The range of ±1000 A/m is more interesting for this type of sensor development due to the better representation of central minima depth and width. The sample annealed with a current of 575 mA met the best conditions and achieved a change in GMI impedance (according to the Equation (1)) of 255.5% for the anisotropy field equal to 47.1 A/m. The change of the GMI parameter (according to Equation (1)) between the local minimum for the zero magnetizing field H and the maximum peak value of the GMI parameter was 202.7%. The 625 mA current annealed sample achieved a greater GMI impedance change ratio (according to Equation (1)) of 299.6%, but also had a greater anisotropy field value of 65.9 A/m. Annealing in the presence of a magnetic field (current flow through the annealed material by the Joule method, resulting in the creation of a transverse field to the direction of current flow) was shown in [50] to improve the noise properties of the material.

High-resolution material testing also showed the exact shape of the impedance curve in the range (−H_k_, +H_k_). This curve has a single minimum, and in the range of weak magnetizing fields may be approximated by a parabola.

### 2.3. Proposed Magnetic Field Sensor Test Stand

**Figure 4:**
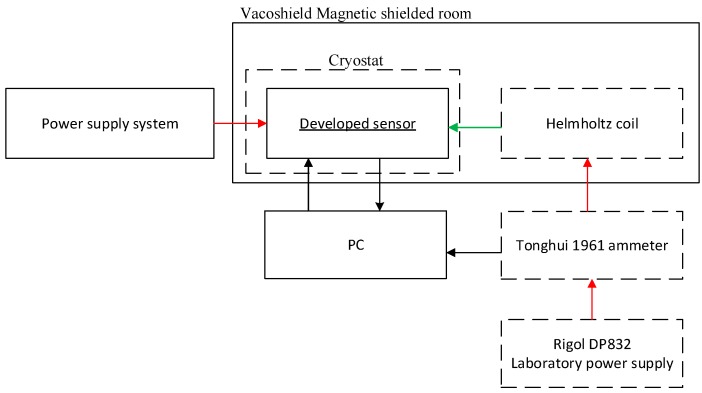
Block diagram of the measurement stand for testing the time and temperature drift of the proposed sensor.

The block diagram of the measurement stand was constructed to determine the sensor processing characteristics as well as the time and temperature drift of the proposed sensor is shown in Figure 4.

Characteristic measurements as well as time and temperature drift tests were carried out in a magnetically shielded chamber. The chamber of the German company Vacumschmelze type Vacoshield MSR (Magnetically Shielded Room) was used. This was made of two layers of MuMetal, 3 mm and 2 mm thick, interleaved with a layer of solid 8 mm aluminum. This configuration allows effective suppression of external magnetic fields in the range from DC up to 1 GHz. The attenuation coefficients declared by the manufacturer are 500 for DC fields, and 30,000 for AC fields with 50 Hz frequency.

Helmholtz coils were used to compensate for the influence of external magnetizing fields. The measured magnetic field H_m_ was generated by a third pair of Helmholtz coils coaxial with the long axis of the GMI sensor’s core. The value of the measured magnetic field H_m_ was calculated from the current flowing through the coils, measured by an ammeter and the coil constant.

Tests on the processing characteristics of the developed sensor consisted of measuring the value of the read voltage U on a shunt resistor, proportional to the compensation current for a given measured magnetic field H_m_. The measured magnetic field was generated in the range of weak magnetic fields (i.e., in the range from −700 A/m to +700 A/m with a step of 2 A/m).

Time drift tests consisted of measuring the indications in the absence of an external magnetic field acting on the sensor for one hour.

For temperature drift testing, an additional cryostat AD07R-40 from PolyScience was used. The cryostat allows the temperature of the liquid medium to stabilize in the range of −40 to 200 °C, with a resolution of 0.01 °C. The sensor processing system was immersed in silicone oil. The tests were carried out in the range from −10 to 70 °C, with a temperature step of 10 °C. 

## 3. Developed Sensor for Measuring Magnetic Fields Using the Magneto–Impedance Phenomenon

### 3.1. The Concept of the Proposed Sensor

The essence of the proposed sensor is to use the properties of the GMI double-peak curve, having the local impedance minimum for the zero external magnetizing field H.

Thus, the operation of the sensor will be based on the compensation of the influence of the external magnetizing field H (measured field) on the active element GMI by creating a compensating field H_com_ with the same value, but the opposite sign.

**Figure 5:**
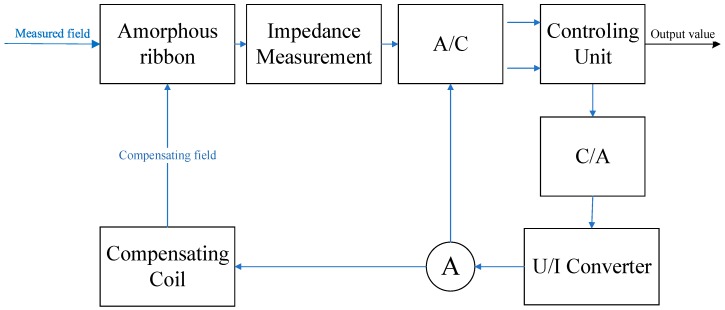
Block diagram of the operation of the proposed sensor.

The simplified diagram of the proposed sensor is shown in Figure 5. The electronic impedance measurement system measures the impedance of the GMI core for a given magnetizing field H. This value is transmitted to the control system using an analog-to-digital converter (A/C). The control system, depending on the stage of sensor operation, sets the next value of the compensating field. The compensating field is set by the control system using a digital-to-analog converter (C/A), a voltage-current converter (U/I), and compensating coils acting on the GMI active element. The ammeter measures the current flowing through the compensation coils, the current value is binarized by the A/C converter, and is calculated by the control system to the value of the compensation field.

**Figure 6:**
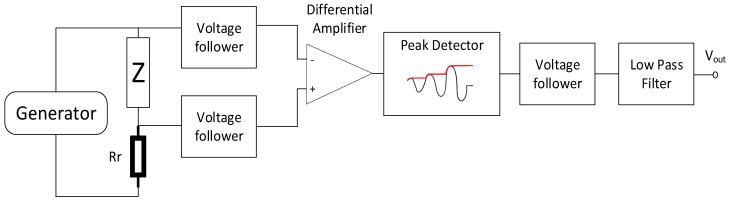
Block diagram of the operation of the impedance measurement block.

Figure 5 illustrates a functional diagram of the proposed sensor and Figure 6 is a block diagram of the electronic impedance measurement system. The sensor works as a compensation sensor, generating its own magnetic field to balance out the external, measured field. The GMI characteristic is utilized as a sensitive null detector, because the center minimum is obtained for a zero external field. It is thus, in essence, a direct measurement method where the measured field is compared with the standard field by the mean of a sensitive null detector (e.g., Wheatstone bridge and similar methods).

The measurement process, the compensation of the measured H_m_ field, will take place in two stages:

- **First stage**—the measurement of the Z characteristic points (H_com_),
- **Second stage**—feedback loop to find the minimum impedance.

**Figure 7:**
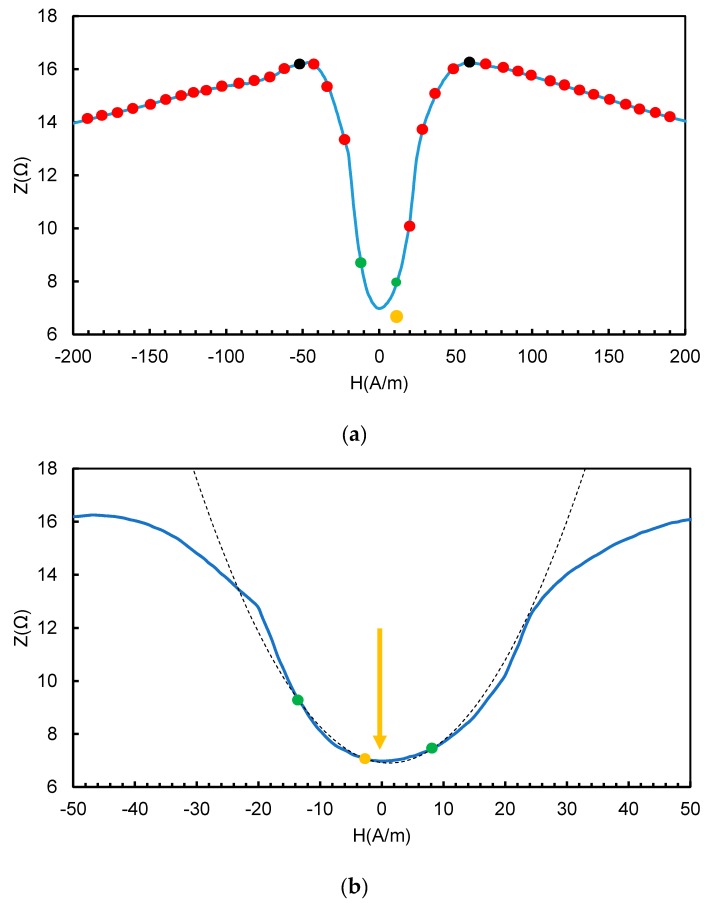
Principle of operation of the proposed sensor: (**a**) Stage I—the measurement of characteristic points, (**b**) Stage II—interpolation of the second-degree curve and compensation of the influence of the external field in order to obtain an indication of the minimum impedance (quasi-Newtonian optimization [51]).

**The first stage** (Figure 7a) consists of measuring the impedance of the GMI active element for subsequent compensation field values (points in Figure 7) in order to detect the local minimum of the two-peak characteristic. In the first stage, the step of changing the compensation field intensity is 5 A/m and is significantly larger than the step of changing the field in the second stage (0.5 A/m). At this stage, characteristic points of the GMI curve are determined from the measured impedance values (red points, Figure 7); the maximums are marked in Figure 7 in black and the minimum is marked in orange. 

**The second stage** (Figure 7b) consists of two phases determining the minimum. Input data for the first phase are:

▪ The value of the compensating field for the minimum measured value and the corresponding impedance value (point marked in orange); and
▪ two points adjacent to this place (points marked in green).

**Figure 8:**
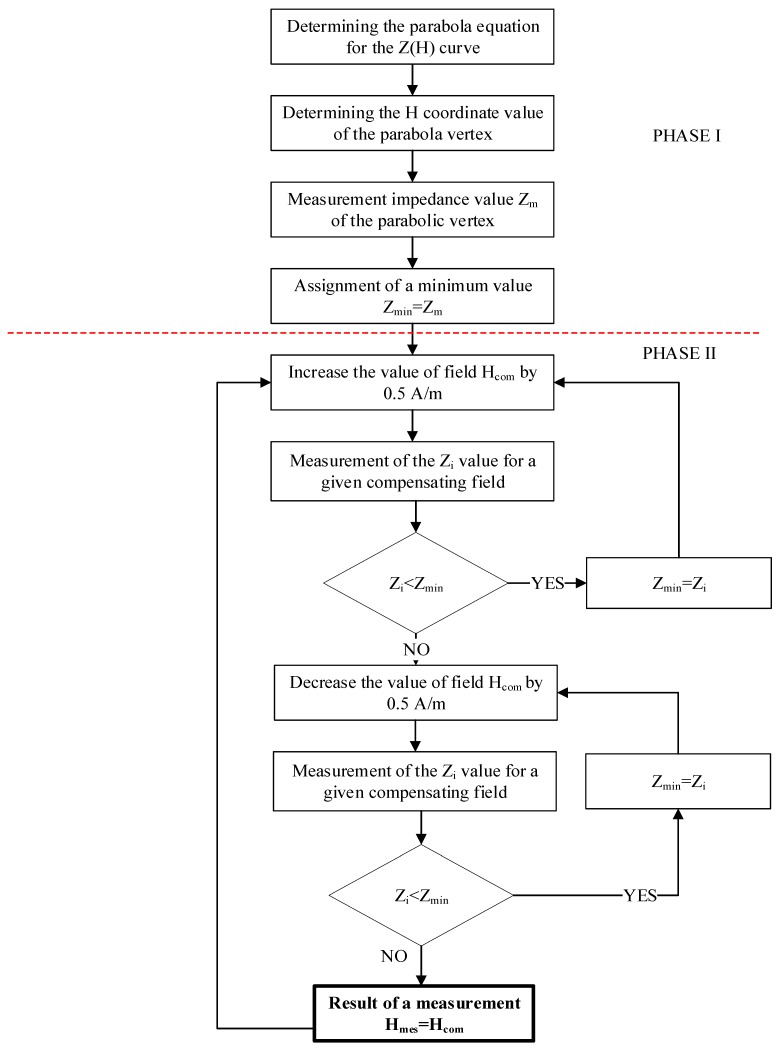
Block diagram of the second stage of the proposed sensor algorithm.

On this basis, the equation of the second-degree curve passing through these points is determined. The curve presented in Figure 7b is an approximation of the actual impedance curve of the GMI conductor for weak magnetizing fields H. The value of the H coordinate of the vertex of the curve is the value from which the program begins operation in the second phase. The algorithm to find the minimum characteristics described above is determined by Newtonian optimization [51]. At this stage, the compensation field setting step value was on the order of 0.5 A/m. The impedance value sought was reached (orange arrow in Figure 7b) when each subsequent change in the compensation field, up or down, gives greater than the minimum impedance results. The block diagram of the sensor operation in the second stage is shown in detail in Figure 8.

### 3.2. The Prototype of the Sensor

**Figure 9:**
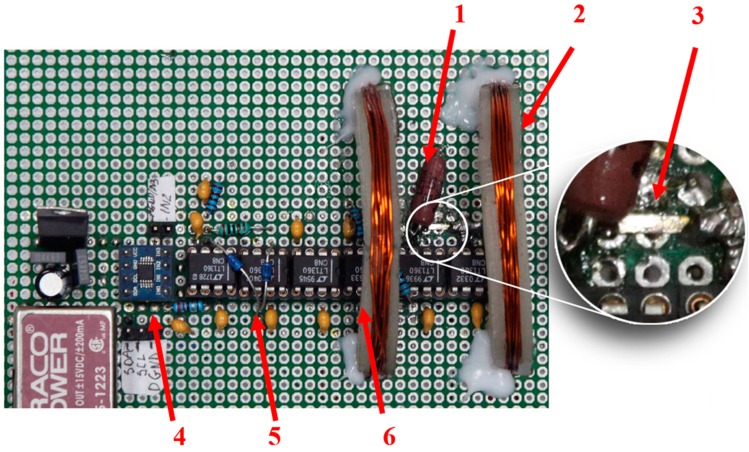
Electronic impedance measurement system. Zoom on the active GMI element: (1) reference resistor; (2) compensation coils; (3) amorphous tape; (4) analog-to-digital converter; (5) peak detector; (6) differential amplifier.

The completed system is shown in Figure 9. In the system, a selected sample of annealed amorphous ribbon was used as the active GMI element. The annealing current optimization process was performed in order to select the most appropriate material for the sensor core. The sample used to build the sensor was annealed with a 575 mA current, which had the most favorable properties as described in Section 2. The ribbon was soldered to the circuit using the Lichtenberg alloy (Bi 50%, Pb 30%, Sn 20%). Due to the Lichtenberg alloy’s low melting point (91.6 °C [52]), soldering with a temperature of 100–110 °C was possible. This soldering temperature is slightly greater than the acceptable ribbon operating temperature (90 °C [53]) and much less than the crystallization temperature of the ribbon (550 °C [53]). This means that soldering did not affect the structure of the material. The sample was placed coaxially between the small compensation coils set in the Helmholtz configuration. Coil carcasses were made in 3D printing technology, enabling the simple and quick production of elements with specific geometric parameters. The coils had n = 27 turns, and the average radius of the coils was 23 mm. According to the Helmholtz coil equation, the coil constant was 840 $\frac{A}{m}/A$. 

The GMI active element operates in a voltage divider configuration with a non-inductive reference resistor. The electronic impedance measurement system consists of a differential amplifier measuring the voltage drop on the GMI active element; a peak detector that rectifies the measured voltage drop; a low-pass filter that eliminates high-frequency interference; and voltage followers that isolate individual functional blocks. The excitation signal had a 10 mA RMS amplitude and a frequency of 10 MHz. The output voltage from the system was measured using a 16-bit ADS1115 analog-to-digital converter. This transducer is characterized by wide possibilities of choosing a measuring range from 256 mV to 6144 V and a sampling rate up to 860 samples per second. A Traco Power TEN 5-1223 converter supplied the entire electronics with 15 V symmetrical voltage. The +5 V supply voltage for the analog-to-digital converter was provided by the LM 7805 voltage regulator.

A voltage-controlled current source in Howland configuration [54] was designed to supply the compensation coils. An operational amplifier LM675T with a maximum operating current limited to 3A was used to build the source. Voltage control was undertaken using a 16-bit LTC2642-16 digital-to-analog converter. This transducer is characterized by a low non-linearity error, the ability to generate symmetrical voltage relative to the system ground, and the ability to communicate with the control unit using the Serial Peripheral Interface (SPI). The proposed system requires a stable reference voltage for proper operation. This condition is met by using the LT6654-2.5 system with a nominal reference voltage V_ref_ = 2.5 V and a voltage generation accuracy of 0.05% of the rated value. The measurement of the current flowing through the compensating coils, proportional to the voltage drop across the shunt resistor, was made using the ADS 1115 analog-to-digital converter.

The myRIO (Reconfigurable Input/Output) platform from National Instruments was chosen as the control unit of the sensor development. The myRIO system is a portable platform for developing embedded control systems. This system is based on a combination of a dual-core processor with a real-time system and an FPGA (field programmable gate array) structure. This architecture allows for very fast acquisition, signal processing, and system response, which is a desirable feature in sensor technology.

**Figure 10:**
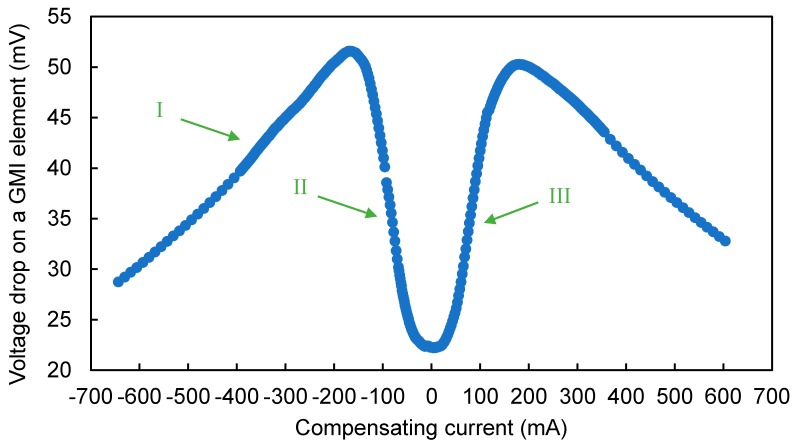
Voltage drop on the GMI sensor’s core as a function of compensating current. I—First rising slope in sequence, II—first falling slope in sequence, III—second rising slope in sequence.

To confirm the correct operation of the developed system, a program for testing the sensor response was proposed, depending on the H_com_ magnetizing field generated by the compensation coils. The program measured the voltage drop on the GMI element and the value of the compensating current. Figure 10 shows the measured characteristics of the GMI element as a function of external field H. The shape of the characteristic corresponds to the shape of the characteristic measured during the tests presented in Section 2.2. This allows for the correct operation of the sensor to be confirmed. In addition, the tests enabled the selection of the optimal measuring ranges and sampling frequencies.

The control system implemented the algorithm proposed in Section 3.1. The program starts its operation by setting the value of the compensation field H_com_ = −700 A/m, and the value of the tape impedance for the set field H_com_ was measured. Then, the compensation field value was increased by 5 A/m. The program ran until three slopes were detected on the Z (H) characteristics:

- First rising slope (I in Figure 10);
- First falling slope (II in Figure 10); and
- Second rising slope (III in Figure 10).

In each loop iteration, it also checked whether the compensation field value H_com_ exceeded the value of the boundary field H_gr_. If the value of H_com_ is greater than H_gr_, the value of H_com_ or H_gr_ is corrected, depending on the current program status. If H_com_ again exceeds the value of H_gr_, it means that the measured value is outside the range of the proposed sensor.

When the search sequence is finished, the approximate value of the H_com_ compensation field is determined. The Z (H) characteristic is interpolated using a second-degree polynomial. Then, the coordinates of the minimum of the obtained quadratic function are calculated. The minimum value on the abscissa is taken as the value of the H_com_ compensation field for the next phase of the program.

Then, the program starts a thorough scan in the “rising field” mode. The value of the H_com_ compensation field in each loop iteration increased by 0.5 A/m. When it is detected that the impedance value does not decrease as the compensation field value changes, the program assumes that the compensation field value H_com_ has been found to balance the measured magnetic field. In this situation, the current flowing through the compensation coils is measured and the H_m_ value is calculated. The measurement result is displayed and the program starts scanning in the “decreasing field” mode. In this mode, the set compensating field is reduced by 0.5 A/m in each step. If it is detected that a change in the compensating field value does not change the impedance of the GMI element, the program assumes that a compensating field value corresponding to the measured field value has been found. Then, the current flowing through the compensation coils is measured and the H_m_ value is calculated. The measurement result is displayed and the program resumes scanning in “rising field” mode. By constantly switching between modes, the sensor responds to fluctuations in the measured magnetic field. If the impedance value exceeds the assumed limit, which will mean a large change in the measured value, the algorithm returns to the first phase of checking (START).

### 3.3. Performance of the Proposed Sensor

The last stage of work on the prototype of the proposed sensor was the measurement of the metrological properties of the system. 

**Figure 11:**
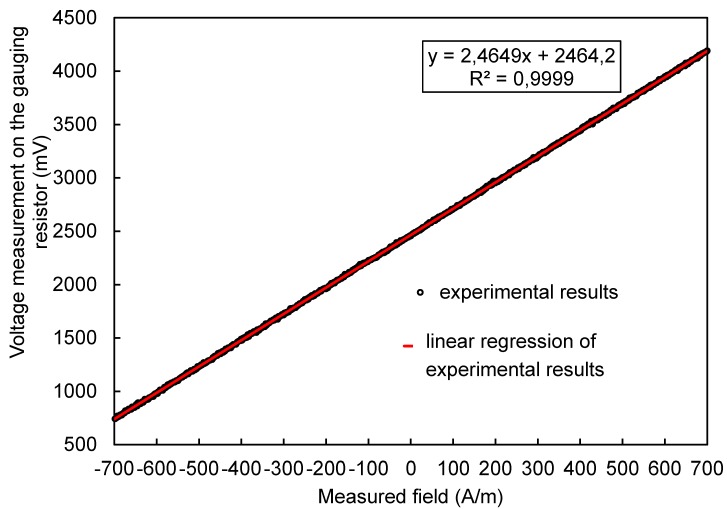
Processing characteristics of the developed sensor.

Figure 11 shows the characteristics obtained along with the linear regression equation for the series of measured values. The linear regression equation was determined using the least-squares method. The high compliance of the fit of the regression were in line with the experimental results (coefficient R^2^ = 0.9999) and the small relative uncertainty value of determining the regression coefficients testify to the linearity of the developed sensor’s characteristic.

By determining the inverse function of the obtained linear regression function, we obtained a relationship that allowed us to determine the value of the measured H_m_ field on the basis of the measured value of the read voltage V on the measuring resistor (mV).

(2) $$H_{m}=\frac{V\left(mV\right)\;-\;2464,2\;mV}{2,4649}\;\frac{A}{m}$$

The uncertainty value was estimated based on the standard deviation of the difference between the measured value and the regression value at a given point. The obtained standard deviation was σ = 6.1 mV. Assuming the extension factor k = 2, the uncertainty of the voltage reading U on the shunt resistor U_v_ = 12.2 mV. This means that the uncertainty of determining the value of the measured field was U_Hm_ = 4.95 A/m, which is 0.7% of the range. This value is lower than the value of typical commercial gaussmeters (2% of the range) [55].

Time drift tests consisted of measuring the indications in the absence of an external magnetic field acting on the sensor for one hour. The resulting time drift value of −5.76 mV/h was less than the value of the estimated uncertainty. This means that the time drift value can be omitted (0.3% of the range).

The measured temperature drift was significantly lower than the estimated uncertainty of indications (0.07% of the range). This demonstrates the possibility of neglecting the impact of temperature drift, especially in comparison with the significant temperature error of commercial hall-effect sensors and magnetoresistive magnetometers [56]. 

## 4. Conclusions

The concept was proposed and a demonstration sensor using the GMI phenomenon was made and tested. The simulation tests carried out enabled a better understanding of the detailed conditions of the sensor operation and the selection of appropriate electronic components. Studies of the demonstrator of the developed sensor for measuring the magnetic field strength confirmed the possibility of the practical use of the sensor for measuring the magnetic field strength in the range of ±700 A/m

It is possible to significantly extend the measuring range of the developed sensor. The estimated expanded uncertainty of the magnetic field strength measurement was 0.7% of the range. It should also be emphasized that the time and temperature drift of the developed sensor was less than the uncertainty of indications, which is very beneficial from the point of view of potential practical applications such as scalable magnetic field sensors, scalable current transducers, or sensors in automotive applications.
